# An Inductorless Self-Controlled Rectifier for Piezoelectric Energy Harvesting

**DOI:** 10.3390/s151129192

**Published:** 2015-11-19

**Authors:** Shaohua Lu, Farid Boussaid

**Affiliations:** University of Western Australia, 35 Stirling Highway, Crawley, Western Australia 6009, Australia; E-Mail: farid.boussaid@uwa.edu.au

**Keywords:** piezoelectric energy harvesting, AC-DC power conversion, SSHI, rectifier

## Abstract

This paper presents a high-efficiency inductorless self-controlled rectifier for piezoelectric energy harvesting. High efficiency is achieved by discharging the piezoelectric device (PD) capacitance each time the current produced by the PD changes polarity. This is achieved automatically without the use of delay lines, thereby making the proposed circuit compatible with any type of PD. In addition, the proposed rectifier alleviates the need for an inductor, making it suitable for on-chip integration. Reported experimental results show that the proposed rectifier can harvest up to 3.9 times more energy than a full wave bridge rectifier.

## 1. Introduction

Harvesting ambient energy provides an opportunity to enable self-powered wireless environmental sensing networks and embedded wearable microelectronic devices [1,2,3]. A number of techniques have been proposed to harvest ambient energy sources such as RF, solar, thermal, and vibration [4]. Among these energy sources, ambient vibrational energy has attracted much attention due to its high energy density (10 to 100’s µW) [5], high integration potential [6,7] and abundance [8,9,10,11]. High efficiency, stand-alone operation and compatibility with semiconductor industry standard CMOS process are important requirements to achieve mass production of piezoelectric energy harvesting systems [12]. Such systems comprise a piezoelectric device (PD) to convert ambient vibrational energy into electrical energy, together with a rectifier. The latter is required because a vibrating piezoelectric device behaves as a capacitive ac current source in parallel with a capacitor and a resistor [13]. The simplest rectifier topology is the full-wave bridge rectifier [7,13,14,15,16]. However, it suffers from low power conversion efficiency because the PD’s internal capacitance is charged and discharged every half cycle [17,18]. In order to overcome this limitation, a high efficiency nonlinear technique Synchronized Switch Harvesting on Inductor (SSHI) was proposed by Guyomar *et al.* [19]. This popular technique uses a series connected switch and inductor in parallel with the piezoelectric device. Every half cycle, when the current produced by PD changes polarity, the switch is closed. As a consequence, the inductor and the PD’s internal capacitance form a LC oscillating network, allowing for the voltage across PD to be naturally inverted. The inversion time corresponds to half the period of the LC oscillating network. However, such a voltage inversion process is limited by the parasitic resistance along the LC oscillating network. The challenges associated to the implementation of such a technique include: (i) the detection of the polarity change of the current produced by the PD [20,21,22,23]; (ii) control of the inversion time given all the different possible values of L and internal capacitance of PD [24]; and (iii) the power required by the control circuits [25,26].

**Figure 1:**
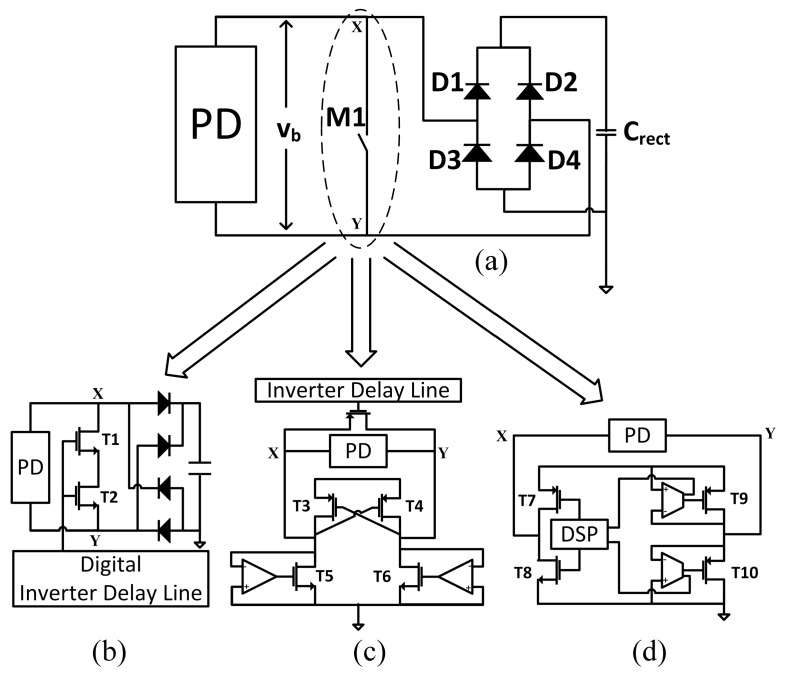
(**a**) Switch-only technique; (**b**–**d**) Existing implementations.

In [27], we proposed a simple yet high efficient SSHI rectifier for piezoelectric energy harvesting. The proposed rectifier monitors the voltages at the two ends of the piezoelectric device (PD) to detect the polarity change of the current generated by the PD. The inversion process of the voltage across the PD is automatically controlled by diodes along the oscillating network. In contrast to prior works, the proposed rectifier combines a number of advantages including high power efficiency, hardware simplicity, standalone operation, but also compatibility with commercially available PDs. However, as the SSHI technique requires a large value for the inductor [24], the previously proposed rectifier is not suitable for CMOS integration. One solution proposed to tackle this issue is to the emulate inductance using a negative impedance [28,29]. In [29], the authors suggest the use of virtual grounded and floating inductors to achieve the required value of inductance. However, these types of implementations remain poor representations of real inductors. In addition, they are large in size, sensitive to component variations and thus difficult to tune. Furthermore, they require the inclusion of additional active elements and external power sources [23]. A switch-only technique solution that alleviates the need for an inductor was proposed in [24] (Figure 1a). This technique puts a switch M1 in parallel with the PD. When the polarity of current produced by the PD changes (at the beginning of every half cycle), switch M1 closes. As a result, the voltage across the PD is discharged without drawing on the energy generated from the PD. [24] proposed an implementation of the switch-only rectifier (Figure 1b), with two transistors implementing switch M1. ON time is controlled by a digital inverter delay line comprising inverter chains separated by multiplexers. By applying different control words to these multiplexers, the delay line can produce different ON times for switch M1. However, the control words are generated externally and need to be tuned for each specific PD so as to calibrate the correct ON time. A similar implementation (Figure 1c) for switch M1 was presented in [30]. The rectifier also uses an inverter delay line to control the ON time of switch M1 but the delay line is not programmable. As a result, the ON time cannot be adjusted to different PDs. In [31], a rectifier (Figure 1d) uses four transistors controlled by custom designed preset dc offset op-amps and DSP to implement switch M1. These custom designed op-amps prevent the transistors turning ON before the current produced by the PD changes polarity. However, the ON time of the transistors cannot accommodate different types of PDs, which would exhibit different internal capacitance values.

To address these limitations, an inductorless self-controlled rectifier is proposed in this paper. The rectifier neither relies on external control signals, nor does it use an inverter chain to control the switch ON time. Furthermore, the rectifier offers high efficiency, low circuit complexity while being fully compatible with CMOS technology as it does not use any inductor.

The paper is organized as follows: Section 2 introduces the electrical model of a piezoelectric device. Section 3 presents the operation and limitations of a conventional full wave bridge rectifier. Section 4 analyses the operation, harvested energy and power loss of the proposed rectifier. Section 5 discusses reported experimental results and provides a performance comparison with prior works. Section 6 concludes the paper.

## 2. Electrical Model of Piezoelectric Device

**Figure 2:**
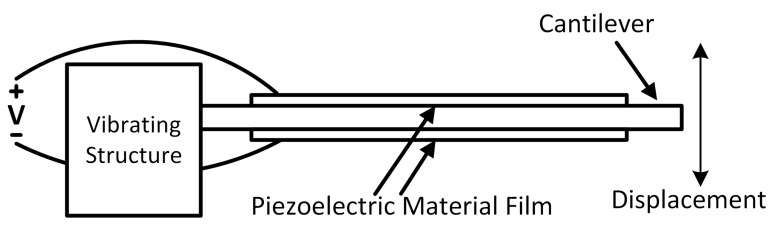
Structure of a piezoelectric device (PD) [36].

**Figure 3:**
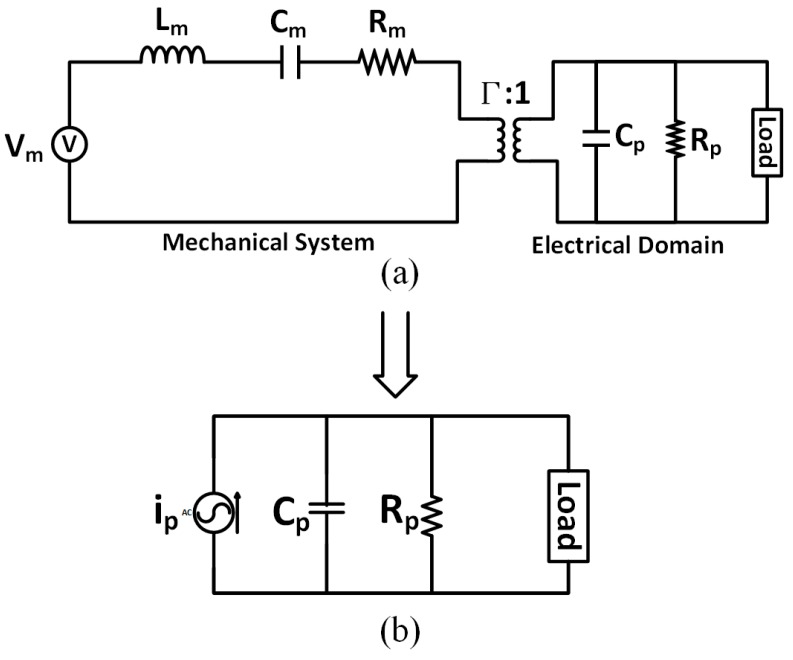
(**a**) Electromechanical model of the piezoelectric device; (**b**) Uncoupled equivalent circuit of a piezoelectric device considering dielectric losses associated to *C_p_*.

Figure 2 shows the structure of a PD composed of a cantilever beam, with two thin piezoelectric material films bonded on the top and bottom surfaces. When subject to vibration, the mechanical stress and strain developed within the piezoelectric material are converted into electrical charge [24]. The electromechanical model (Figure 3a) of a piezoelectric device can be represented as coupling a mechanical system to electrical domain through a perfect transformer [32]. The primary side of the transformer represents the mechanical system, with *V_m_* representing the input vibration, *L_m_* the mass, *C_m_* the mechanical stiffness and R_m_ the mechanical loss. The secondary side of the transformer represents the electrical load and characteristics of the piezoelectric device, with *C_p_* representing its internal capacitance and *R_p_* representing its internal resistance. Parameter Γ (Figure 3a) is a measure of the electromechanical coupling of the piezoelectric element. This parameter provides a measure of the efficiency of energy conversion between mechanical and electrical domains. Because most piezoelectric devices have low coupling coefficients, damping from the electrical side can often be neglected [33,34,35]. As a result, the equivalent circuit of the piezoelectric device can often be simplified to a current source *i_p_* in parallel with the internal capacitance *C_p_* (Figure 3b) [33,34,35]. This uncoupled model assumes that the internal current source is mostly unaffected by the external load. This is equivalent to assuming that the vibration amplitude is independent of the external load [33,34,35]. For most practical applications, the harvest power can be boosted significantly by the interface circuits before *i_p_* changes notably [34]. As a result, and for purposes of simplicity and clarity, the uncoupled equivalent circuit model (Figure 3b) is widely used and adopted by all prior works (Table 1). Such a circuit often also takes into account the dielectric losses associated to *C_p_*, by including a parallel resistor *R_p_* (Figure 3b), whose value is usually very large (MΩ) [33,34,35]. This high internal resistance restricts the amount of output current. Another non-ideal characteristic of the PD is its low output voltage when the input vibration level is low. This makes it difficult to design an efficient rectifier since the diodes in the rectifier usually have non-zero turn on voltages [24].

## 3. Conventional Rectifier

**Figure 4:**
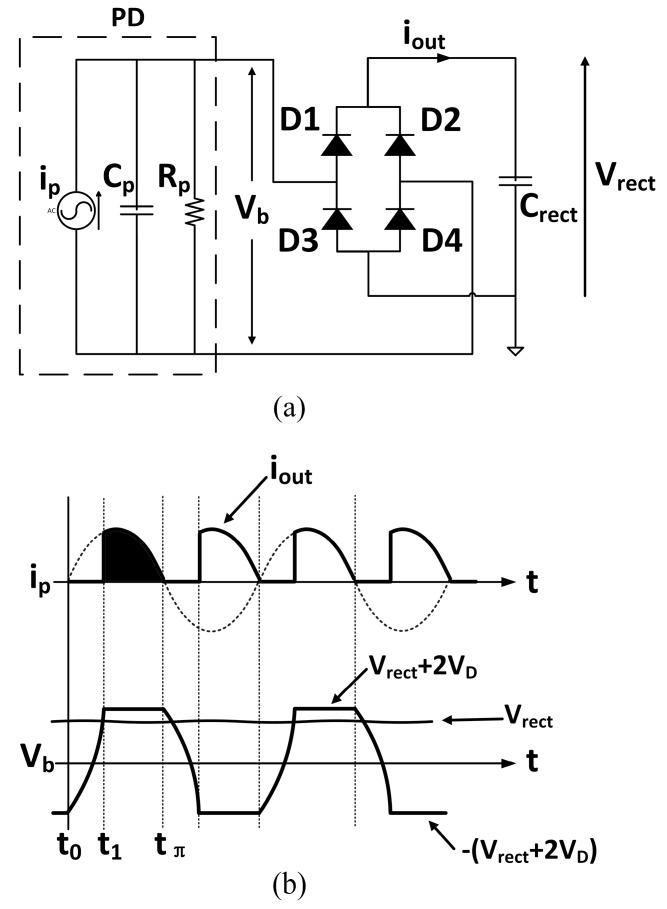
(**a**) Conventional full wave bridge rectifier; (**b**) Input and output waveforms.

The conventional full wave bridge rectifier (Figure 4a) is traditionally used to convert the AC output voltage of a piezoelectric device into a DC voltage. The output capacitor *C_rect_* is chosen to be large enough to make the output voltage *V_rect_* constant. The corresponding input and output waveforms of current and voltage are shown in Figure 4b. The operation of the full wave bridge rectifier can be divided into two regions: (i) from time t_0_ to t_1_, voltage *V_b_* is smaller than the output voltage *V_rect_* (voltage across the capacitor *C_rect_*) and all diodes are reverse biased. Current i_p_ cannot thus flow to the output capacitance *C_rect_.* On the other hand, the internal capacitance *C_p_* is charged up; (ii) from time *t*_1_ to *t*_π_, *V_b_* is first equal then higher than the value of output voltage. Therefore current i_p_ flows into *C_rect_* until current *i_p_* changes polarity. In the first region, the energy generated by PD is dissipated on the internal capacitance *C_p_* and resistance *R_p_*. As a result, the energy generated in this region cannot be harvested. The energy harvested in the second region is represented by the shaded area in Figure 4b. To calculate the harvested energy, one needs first to determine the average output current *i_out_*, which can be expressed as:

(1) $$\begin{array}{cc} i_{out,average} & =\frac{1}{t_{\pi }}\left[\int_{t_{1}}^{t_{\pi }}I_{p}\text{sin}\omega tdt-\frac{V_{rect}+2V_{D}}{R_{p}}\left(t_{\pi }-t_{1}\right)\right]\\ & =\frac{1}{t_{\pi }}\left[I_{p}\left(-\frac{\text{cos}\omega t_{\pi }}{\omega }+\frac{\text{cos}\omega t_{1}}{\omega }\right)-\frac{V_{rect}+2V_{D}}{R_{p}}\left(t_{\pi }-t_{1}\right)\right] \end{array}$$

where *I_p_* and *ω* are the amplitude and angular frequency of input current *i_p_*, respectively. The energy harvested by the full wave bridge rectifier is thus:

(2) $$P_{full\_bridge}=\frac{V_{rect}}{t_{\pi }}\left[I_{p}\left(-\frac{\text{cos}\omega t_{\pi }}{\omega }+\frac{\text{cos}\omega t_{1}}{\omega }\right)-\frac{V_{rect}+2V_{D}}{R_{p}}\left(t_{\pi }-t_{1}\right)\right]$$

Since *ωt*_π_ = π, hence:

(3) $$P_{full\_bridge}=\frac{V_{rect}}{t_{\pi }}\left[\frac{I_{p}}{\omega }\left(1+\text{cos}\omega t_{1}\right)-\frac{V_{rect}+2V_{D}}{R_{p}}\left(t_{\pi }-t_{1}\right)\right]$$

where:

(4) $$t_{1}=\frac{{\text{cos}}^{-1}\left(1-\frac{2\left(V_{rect}+2V_{D}\right)\omega C_{p}}{I_{p}}\right)}{\omega }$$

The maximum power harvested by the full wave bridge rectifier is obtained when [32]:

(5) $$V_{rect}=\frac{I_{p}}{\omega C_{p}2}-V_{D}$$

with the maximum power given by:

(6) $$P_{full\_bridge,\max}=C_{p}{\left(\frac{I_{p}}{\omega C_{p}}-2V_{D}\right)}^{2}\frac{\omega }{2\pi }$$

## 4. Proposed Inductorless Self-Controlled Rectifier

### 4.1. Operation Principle

The major limitation of the full wave bridge rectifier was shown to be with the fact that the charge generated by the PD cannot be harvested when the output voltage is higher than the voltage across the PD (Figure 4b). This is because the internal capacitance of the PD needs to be first discharged and then charged again at a voltage higher than the output voltage for the generated charge to flow to the output. To address this issue, we propose an inductorless self-controlled rectifier which can short both ends of the PD to ground at the beginning of every half cycle. As a result, the energy generated by the PD only needs to charge up the internal capacitance, thereby saving significant energy.

**Figure 5:**
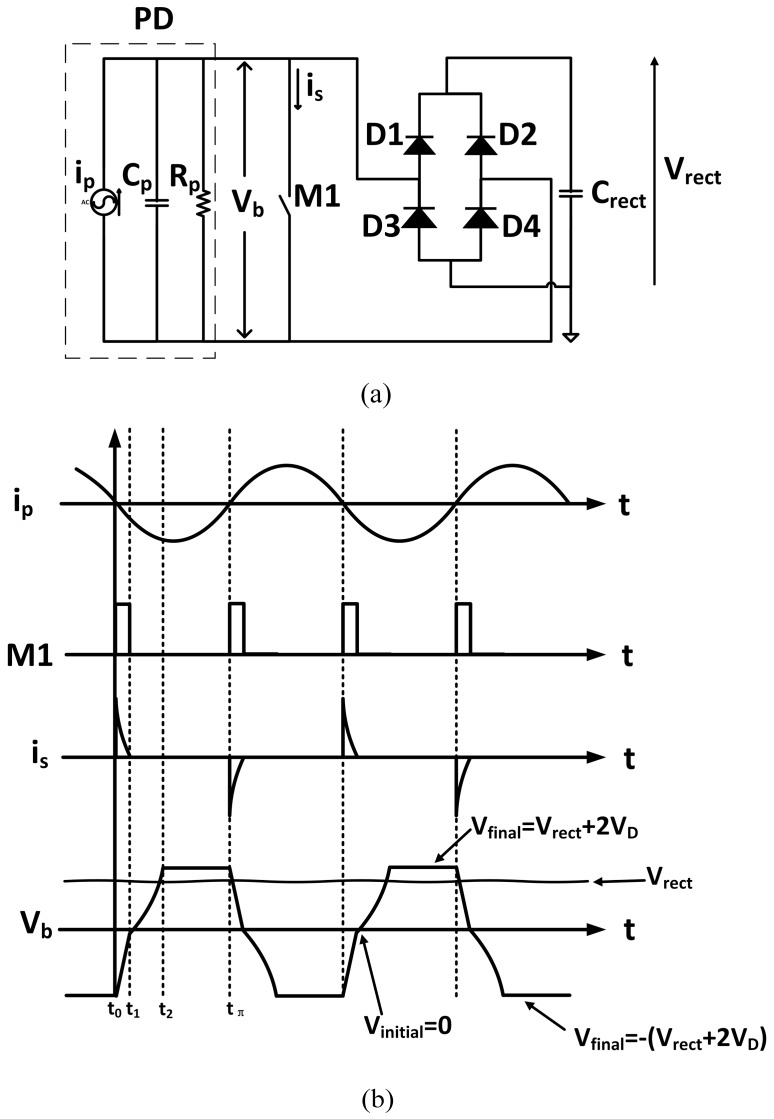
(**a**) Proposed rectifier scheme; (**b**) Associated voltage and current waveforms.

Figure 5a shows the proposed rectifier, which has a switch M1 connected in parallel with the PD. At the beginning of every half cycle, the current *i_p_* produced by the PD changes polarity, the switch M1 is turned ON. As a consequence, the charges stored in the PD’s internal capacitance *C_p_* are immediately discharged to ground through switch M1. As soon as *C_p_* becomes fully discharged, switch M1 is turned OFF. As a result, current source *i_p_* only needs to charge up *C_p_* from ground to ±(V_rect_ + 2V_D_), before the current starts flowing to the output. The corresponding voltage and current waveforms are shown in Figure 5b. Every half cycle, the total charge delivered to the output by the proposed rectifier is the total charge produced by the PD minus the charge loss on the internal components *C_p_* and *R_p_* of the PD and on the diodes. The latter is given by:

(7) $$Q_{harvest}=Q_{total}-Q_{loss,C_{p}}-Q_{loss,R_{p}}$$

The total charge produced by the PD every half cycle is thus:

(8) $$Q_{total}=2C_{p}V_{p}$$

where V_p_ is the open circuit voltage of the PD, which is given by:

(9) $$V_{p}=\frac{I_{p}}{\omega C_{p}}$$

Since the internal capacitance *C_p_* is discharged through M1 in a very short time interval [*t*_0_, *t*_1_] compared to the half cycle of current *i_p_*, the charge lost in time interval [*t*_0_, *t*_1_] can be neglected. The charge lost on the internal capacitance *C_p_* from t_1_ to t_2_ is thus:

(10) $$\begin{array}{cc} Q_{loss,C_{p}} & =\left(V_{final}-V_{initial}\right)C_{p}\\ & =\left[\left(V_{rect}+2V_{D}\right)-0\right]C_{p}\\ & =\left(V_{rect}+2V_{D}\right)C_{p} \end{array}$$

The charge lost on the internal resistance *R_p_* can be divided into two regions: (1) *V_b_* is smaller than output voltage *V_rect_* from *t*_1_ to *t*_2_; (2) *V_b_* is greater than *V_rect_* from *t*_2_ to *t*_π_. In the first region, the charge lost on *R*_p_ is:

(11) $$Q_{loss,R_{p},region1}=\int_{t_{1}}^{t_{2}}i_{R_{p},region1}dt=\int_{t_{1}}^{t_{2}}\frac{v_{b}}{R_{p}}dt$$

where:

(12) $$\begin{array}{cc} v_{b} & =\frac{1}{C_{p}}\int_{t_{1}}^{t}I_{p}\text{sin}\omega tdt+v_{b}\left(t_{1}\right)\\ & =\frac{I_{p}}{\omega C_{p}}\left(\text{cos}\omega t_{1}-\text{cos}\omega t\right)+v_{b}(t_{1})\\ & =V_{p}\left(\text{cos}\omega t_{1}-\text{cos}\omega t\right)+v_{b}\left(t_{1}\right) \end{array}$$

Since *V_b_*(*t*_1_) = *V_initial_* = 0 and *ωt*_1_ is approximately equal to 0, hence:

(13) $$v_{b}=V_{p}\left(1-\text{cos}\omega t\right)$$

Bringing *V_b_* back to Equation (11):

(14) $$\begin{array}{cc} Q_{loss,R_{p},region1} & =\int_{t_{1}}^{t_{2}}\frac{V_{p}\left(1-\text{cos}\omega t\right)}{R_{p}}dt\\ & =\frac{V_{p}\left[\left(t_{2}-t_{1}\right)\omega +\text{sin}\omega t_{1}-\text{sin}\omega t_{2}\right]}{\omega R_{p}}\\ & =\frac{V_{p}\left(\omega t_{2}-\text{sin}\omega t_{2}\right)}{\omega R_{p}} \end{array}$$

Taking the boundary conditions for *V_b_* at time *t*_2_:

(15) $$v_{b}\left(t_{2}\right)=V_{rect}+2V_{D}=V_{p}\left(1-\text{cos}\omega t_{2}\right)$$

Hence:

(16) $$t_{2}=\frac{{\text{cos}}^{-1}\left(1-\frac{V_{rect}+2V_{D}}{V_{p}}\right)}{\omega }$$

In second region [*t*_2_, *t*_π_], the charge lost on *R_p_* is:

(17) $$Q_{lost,R_{p},region2}=\frac{V_{rect}+2V_{D}}{R_{p}}\left(t_{\pi }-t_{2}\right)$$

where:

(18) $$t_{\pi }=\frac{\pi }{\omega }$$

Finally, the harvested power is thus:

(19) $$P_{harvest}=2fV_{rect}Q_{harvest}$$

with the power losses due to diodes being approximately equal to:

(20) $$P_{lost,diodes}\approx 2V_{D}i_{out,average}$$

### 4.2. Implementation

**Figure 6:**
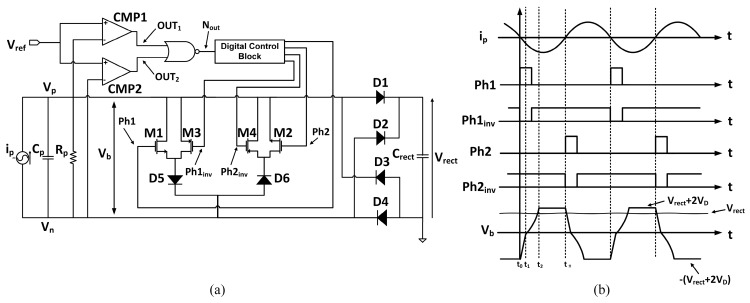
(**a**) Implementation of proposed self-controlled inductorless rectifier; (**b**) associated voltage and current waveforms.

The implementation of the proposed inductorless self-controlled rectifier is given in Figure 6. To detect the polarity change of *i_p_*, the voltages *V_p_* and *V_n_* are compared with a reference voltage *V_ref_*, chosen to be slightly higher than the negative value of diodes’ forward voltage −*V_D_*. When *i_p_* is positive (before *t*_0_) and diodes 1 and 4 are ON, *V_p_* is close to *V*_rect_ + *V_D_* and *V_n_* is close to −*V_D_* but lower than *V_ref_*. Comparators CMP1 and CMP2 evaluate *V_p_* and *V_n_* against *V_ref_*. As a consequence, the outputs of comparators OUT_1_ and OUT_2_ are low and high respectively, since *V_p_* is higher than *V_ref_* and *V_n_* is lower than *V_ref_*. As a result, the output of the NOR gate *N_out_* is low. Once current *i_p_* becomes negative at *t*_0+_, the voltage *V_n_* increases and reaches the value of *V_ref_*. As a result, OUT_2_ toggles from high to low while OUT_1_ stays low.

**Figure 7:**
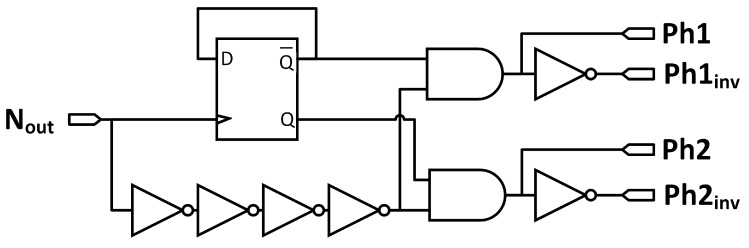
Digital control block.

This makes the output *N_out_* of the NOR gate toggles from low to high. Therefore, a pulse is generated for detecting the polarity change of *i_p_*. A similar process occurs when *i_p_* changes polarity again. Signal *N_out_* is then passed to the digital control block (Figure 7) to generate two polarity change detecting signals. These signals control the ON and OFF time of the transistors M1–M4.

At time *t*_0_, *i_p_* changes polarity from positive to negative, the signals *Ph1* and *Ph1_inv_* are firstly generated to turn on transistors M1 and M3. At this time, a discharging path is formed by transistors M1, M3 and diode D5. As a result, the charge stored in internal capacitance *C_p_* is discharged through this path. This discharging process lasts from t_0_ to t_1_ and is automatically terminated by diode D5. Subsequently, the current *i_p_* charges *C_p_* from about –*V_D_* to (*V_rect_* + 2*V_D_*) in time interval [*t*_1_, *t*_2_] and then delivers power to the output. When *i_p_* changes polarity again from negative to positive, a similar process occurs for transistors M2, M4 and diode D6.

Figure 7 shows the implementation of the digital control block with input *N_out_* and outputs *Ph1*, *Ph1_inv_*, *Ph2* and *Ph2_inv_.* Signal *N_out_* is used as the CLK input for the positive edge triggered D flip-flop. With the D flip-flop’s complemented output connected to its D input, outputs Q and Q bar have both a frequency that is half that of the input *N_out_* signal. They also have the same pulse width, which is double that of the *N_out_* signal. Giving that outputs Q and Q bar are ANDed with the delayed version of *N_out_* signal, signals *Ph1* and *Ph2* have the same pulse width than input signal *N_out_* and half its frequency. *Ph1_inv_* and *Ph2_inv_* are inverted forms of signals *Ph1* and *Ph2.*

## 5. Experimental Results and Discussion

The proposed rectifier was demonstrated using ultra-low power off-the-shelf ICs. Two ultra-low power comparators (LTC1540 Linear Technology, Milpitas, CA, USA, 680 nA max quiescent supply current) were used to implement comparators CMP1 and CMP2 (Figure 6a). Standard 4000 series CMOS gates with low input current leakage were used to build NOR gate and the frequency divider (Figure 7). Switches in the discharging path were implemented using two types of MOSFETs (VN0104 and VP0104 Microchip, Chandler, AZ, USA), with on resistance of 3 Ω and 11 Ω for a gate voltage of 5 V, respectively. All diodes in the proposed rectifier are Schottky diodes (BAT54 Fairchild Semiconductor, San Jose, CA, USA).

**Figure 8:**
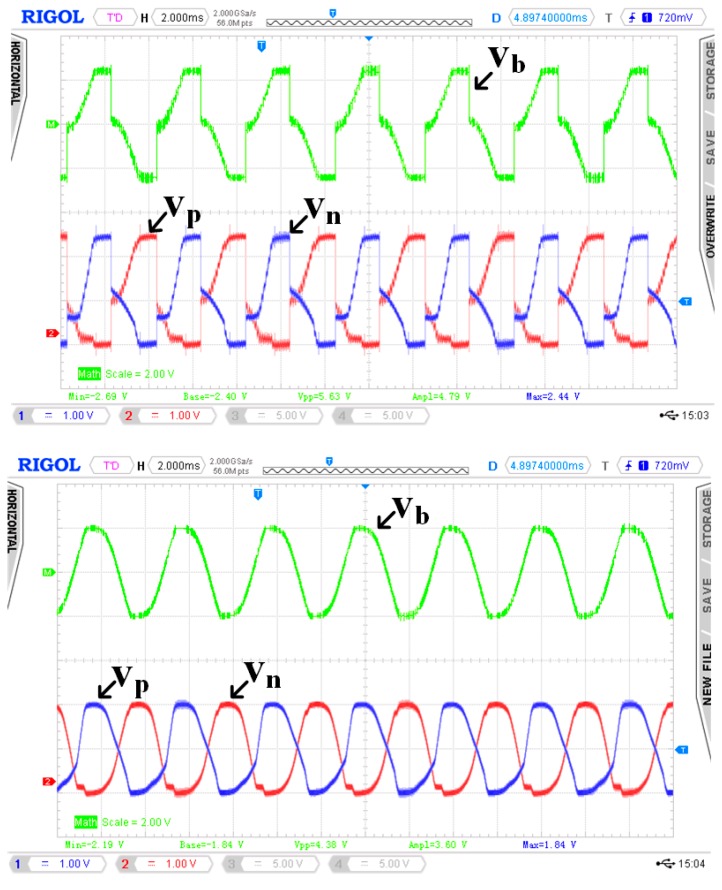
Measured waveforms of the output voltages across PD for the proposed rectifier (**top**) and the full wave bridge rectifier (**bottom**).

Experiments were carried out to evaluate the performance of the proposed rectifier implementation. In our experimental Labworks setup, only the vibration frequency and acceleration amplitude can be set. The PD (V21B Mide Technology, Medford, OR, USA) is screwed on an aluminium plate, which is mounted (using screws) on an electrodynamic shaker (ET-126B-4 Labworks Inc., Costa Mesa, CA, USA). The shaker is driven by a sine wave generator (SG-135 Labworks Inc., Costa Mesa, CA, USA) amplified through a power amplifier (PA-138 Labworks Inc., Costa Mesa, CA, USA). The output signal of a vibration acceleration sensor (model J352C33 PCB Piezotronics, Depew, NY, USA), fixed on the shaker plate, is fed to a controller unit, which ensures that the acceleration amplitude of the shaker plate is kept constant, regardless of the electromechanical feedback introduced by the electrical load. When the PD vibrates at or close to its resonant frequency, the current generated by the PD is proportional to the acceleration [33]. By measuring the open-circuit voltage *V_p_*, it is then possible to deduce the current *I_p_* using Equation (9). The acceleration can be adjusted on the signal wave generator (SG-135 Labworks Inc., Costa Mesa, CA, USA) to achieve the required values of *V_p_* and thus *I_p_*. When setting the vibration frequency to 246 Hz and the acceleration to 0.9 g (1 g = 9.8 m/s^2^) on the sine wave generator (SG-135 Labworks Inc., Costa Mesa, CA, USA), the amplitude of the resulting current generated by the PD is about 390 µA. Figure 8 shows the resulting oscilloscope waveforms for the voltage across PD when attached to the proposed rectifier and full wave bridge rectifier, respectively. Measurements showed that the PD’s open circuit voltage is 1.7 V and its open-circuit frequency is 246 Hz. The obtained waveforms are consistent with the described operations of the proposed rectifier scheme shown in Figure 6b. The proposed rectifier is seen to automatically discharge the voltage across PD to ground at the beginning of every half cycle. As can be observed in Figure 8 (top), the voltage V_b_ does not fully discharge to 0. This is due to the forward voltage of diodes and parasitic resistance along the discharging path.

**Figure 9:**
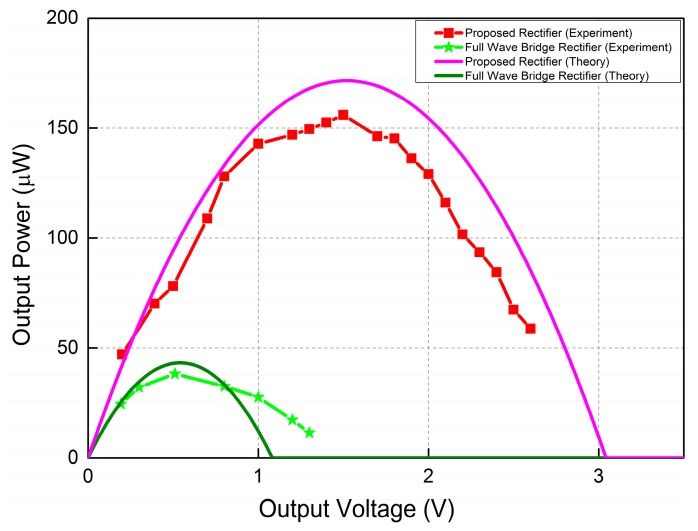
Measured output power.

Figure 9 reports the measured output power as a function of the output voltage. The curve at the bottom with star symbols is the output power of a full wave bridge rectifier. Note that the maximum output of 40 µW is achieved for an output voltage of 0.51 V and diodes’ forward voltage of 0.3 V. The curve with square symbols is the output power of the proposed rectifier. The maximum power of 156 µW is here achieved when the output voltage reaches 1.5 V. Reported measurements show that the proposed rectifier can improve the harvested power by 3.9 times compared to the full wave bridge rectifier.

**Figure 10:**
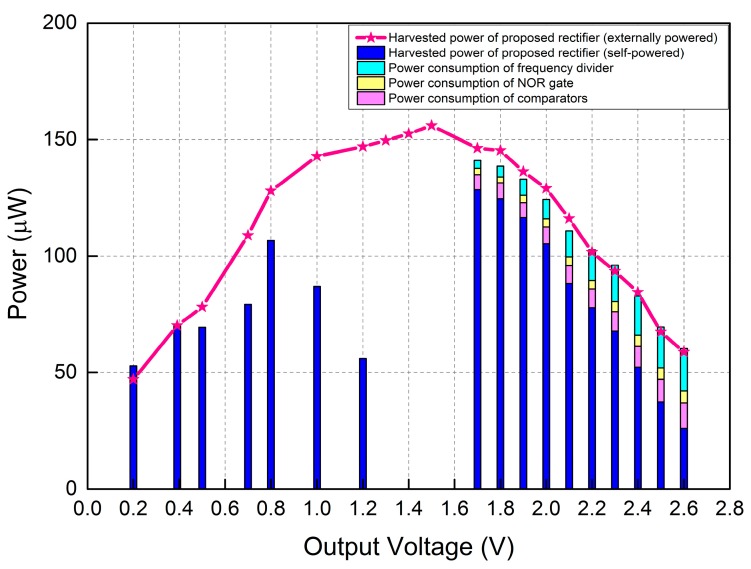
Power measurements of control circuit elements against output power of proposed rectifier.

Figure 10 reports the measured output power of the proposed rectifier together with the power consumption of the control circuit elements. Experimental results are given for the case where the proposed rectifier is self-powered by *V_rect_* but also for the case where it is externally powered. It can be seen from Figure 10 that: (i) for output voltages *V_rect_* < 1.7 V, the proposed rectifier works as a passive rectifier since *V_rect_* is less than the minimum positive voltage supply requirement for the comparators and (ii) for output voltage *V_rect_* > 1.7 V, the control circuits are enabled and the output power is greatly increased, while a certain amount of harvested energy is used to power the control circuits. The power consumption of control circuit elements was obtained by measuring the average supply current and voltage across each component using a Rigol DM3068 digital multimeter. The results show that the difference in output power between externally powered and self-powered rectifiers corresponds to the total power consumption of the control circuits. As observed in Figure 10, when the output voltage *V_rect_* is less than 0.4 V, the output power is the same for both externally powered rectifier and self-powered rectifiers. This is because in this range, the voltage across the PD is smaller or slightly over the forward voltage of diodes along the discharging path. As a result when the switch is turned ON, the charge stored in the internal capacitance is blocked by diodes along the discharging path and cannot flow to ground.

Table 1 compares the performance of the proposed rectifier with reported inductorless rectifiers for piezoelectric energy harvesting [24,30,31]. As in prior works, we compare the proposed rectifier against a conventional full bridge rectifier using the same PD operated in the same experimental conditions. This is done because the actual harvested power cannot be used as a performance metric given that the input power provided by the PD is a function of its mechanical properties, dimensions, resonant frequency, internal capacitance and resistance but also on experimental conditions (e.g., type of shakers, PD positioning onto the shaker). Each of these parameters greatly affects the power generated by the PD. The rectifiers in [24,30,31] and this work both use piezoelectric cantilever beams as PDs for testing the performance of the rectifiers. This type of PD is not suitable for broadband piezoelectric energy harvesting when the ambient vibration frequencies cover a wide range. This is because the PD can only reach its maximum output when it vibrates at or close to its resonant frequency. The resonant frequency of the PD can be adjusted by attaching a proof mass. The works in [30,31] use equivalent circuits to mimic the PD. The amplitude *I_p_* of current produced by PD is proportional to the vibration amplitude. Getting a high value of *I_p_* results in a PD producing more energy, as shown in Equation (8). However, the harvested energy does not depend only on the amplitude *I_p_* but also on the internal capacitance *C_p_*. When two different PDs produce the same amplitude *I_p_*, the one with the smaller internal capacitance *C_p_* can be used to harvest more energy, as shown in Equation (10). Therefore, the internal capacitance of the PD should be made as small as possible. The forward voltage of diodes is a major source of power loss for rectifiers. The voltage drop across diodes should be as low as possible to reduce their energy loss. Schottky diodes were used for [30,31] and this work because they have lower voltage drops and higher switching speed. In addition, Schottky diodes can be implemented in standard semiconductor industry CMOS process [37]. To get a voltage drop lower than that of Schottky diodes, authors in [30,31] implemented custom designed op-amp based diodes. The custom designed op-amps were designed to have a preset dc offset voltage that prevents toggling before the polarity change of the current produced by the PD. This was achieved by applying different aspect ratios to op-amp’s input transistors. However, the mismatch of the preset offset voltages associated to process variations degrades the performance of the rectifier [30]. Furthermore, these custom designed op-amps require constant current input, as well as a custom designed supply independent bias circuit to act as the start-up circuit for the rectifier. Due to the use of discrete components for control circuits in this work, the maximum quiescent current consumption is much higher than other works. However, it can significantly be lowered once the complete rectifier is integrated onto a chip. Eliminating the requirement of inductor (external or internal) makes the proposed rectifier fully compatible with semiconductor industry standard CMOS process. The efficiency of the rectifier, defined as the ratio of the measured maximum output power to the theoretical maximum power (Figure 9), is 91.23%. The ratio of measured maximum harvested power of the proposed rectifier to that of the full wave bridge rectifier was found to be 3.9 times. The proposed rectifier shows several advantages over other designs listed in Table 1, including efficiency, standalone operation, circuit simplicity, compatibility with standard semiconductor industry CMOS process and compatibility with different types of PDs.

**Table 1:** Performance comparison with inductorless rectifiers for piezoelectric energy harvesting.

Publication	[24]	[30]	[31]	This Work
Technology	Integrated	Integrated	Integrated	Discrete
Piezoelectric Device (PD)	Mide Technology (V22B)	Equivalent Circuit	Equivalent Circuit	Mide Technology (V21B)
Amplitude I_p_ of current produced by PD	63 µA	88 µA	94 µA	136.64 µA
Internal Capacitance C_p_	18 nF	25 nF	25 nF	52 nF
Vibration Frequency	225 Hz	200 Hz	200 Hz	246 Hz
Diode Forward Voltage	0.05 V	0.01 V	0.01 V	0.1 V
Start-up circuit	No	Yes	Yes	No
Max Quiescent Current Consumption	>220 nA	180 nA	>180 nA	4900 nA
External Inductor Required	No	No	No	No
Efficiency	Not shown	90%	91.2%	91.23%
Performance compared with a Full Wave Bridge Rectifier	1.9 times	3.4 times	3.5 times	3.9 times

## 6. Conclusions

An inductorless self-controlled rectifier for piezoelectric energy harvesting has been presented in this paper. It overcomes the limitations of existing high performance inductorless rectifiers, which rely on complex DSP and or external control signal circuitry to detect the occurrence of the polarity change of the PD output current but also to control the time required to discharge the internal capacitance of the PD. Furthermore, the proposed rectifier alleviates the need for an inductor, making it suitable for chip integration. This was achieved by using two voltage comparators at the two ends of the PD to monitor polarity change of its output current. The switch ON time is automatically controlled by the diodes along the discharging path. Although the proposed inductorless self-controlled rectifier was implemented using discrete components, it still can improve the harvested energy by up to 3.9 times compared to that of a conventional full wave bridge rectifier.
